# HBV-Specific CD8+ T-Cell Tolerance in the Liver

**DOI:** 10.3389/fimmu.2021.721975

**Published:** 2021-08-06

**Authors:** Ian Baudi, Keigo Kawashima, Masanori Isogawa

**Affiliations:** ^1^Department of Virology and Liver Unit, Nagoya City University Graduate School of Medical Sciences, Nagoya, Japan; ^2^Research Center for Drug and Vaccine Development, National Institute of Infectious Diseases, Tokyo, Japan

**Keywords:** T cell exhaustion, liver tolerance, co-inhibitory signaling, metabolic regulation, intrahepatic antigen recognition, interferon signaling, hepatitis B virus

## Abstract

Hepatitis B virus (HBV) remains a leading cause of liver-related morbidity and mortality through chronic hepatitis that may progress to liver cirrhosis and cancer. The central role played by HBV-specific CD8+ T cells in the clearance of acute HBV infection, and HBV-related liver injury is now well established. Vigorous, multifunctional CD8+ T cell responses are usually induced in most adult-onset HBV infections, while chronic hepatitis B (CHB) is characterized by quantitatively and qualitatively weak HBV-specific CD8+ T cell responses. The molecular basis of this dichotomy is poorly understood. Genomic analysis of dysfunctional HBV-specific CD8+ T cells in CHB patients and various mouse models suggest that multifaceted mechanisms including negative signaling and metabolic abnormalities cooperatively establish CD8+ T cell dysfunction. Immunoregulatory cell populations in the liver, including liver resident dendritic cells (DCs), hepatic stellate cells (HSCs), myeloid-derived suppressor cells (MDSCs), may contribute to intrahepatic CD8+ T cell dysfunction through the production of soluble mediators, such as arginase, indoleamine 2,3-dioxygenase (IDO) and suppressive cytokines and the expression of co-inhibitory molecules. A series of recent studies with mouse models of HBV infection suggest that genetic and epigenetic changes in dysfunctional CD8+ T cells are the manifestation of prolonged antigenic stimulation, as well as the absence of co-stimulatory or cytokine signaling. These new findings may provide potential new targets for immunotherapy aiming at invigorating HBV-specific CD8+ T cells, which hopefully cures CHB.

## Introduction

Hepatitis B virus (HBV) chronically infects more than 250 million people worldwide, which is more than seven times the number of the human immunodeficiency virus (HIV) (1). Chronic hepatitis B (CHB) accounted for over 800,000 deaths in 2015, rivaling HIV (2). Of the estimated 250 million chronic HBV (CHB) carriers worldwide, treatment is indicated in just a small fraction (10-30%) (1). Moreover, although current HBV therapies like nucleos(t)ide analogs (NAs) can effectively suppress viral replication, they are incapable of directly targeting the stable episomal HBV reservoir, the covalently closed circular DNA (cccDNA) (2). CHB patients remain at risk of developing liver cirrhosis and cancer despite available potentially life-long and non-curative treatment (3, 4). This situation justifies an urgent need for more effective HBV therapies.

The central role played by T cell responses in the control of HBV infection is now well recognized (5–7). Immunocompetent human adults readily clear acute HBV infection, up to 95% of the time (8). T cell responses behind the transient, self-limited infections are often described as strong and polyclonal (9–11). In experimentally infected chimpanzees, depletion of CD8+ T cells at the peak of viremia delays viral clearance until the T cells return, providing the most definitive evidence that HBV clearance is largely mediated by virus-specific CD8+ T cells. Meanwhile, CD4+ T cells aid the activation and maintenance of the CD8+ T cell responses in addition to triggering HBV-specific humoral responses that prevent viral dissemination (12, 13). In CHB patients, T cell responses are quantitively weak, and if present, functionally impaired (9, 14). It has become evident that multiple factors contribute to T cell dysfunction, but immunological events in the liver appear particularly important to establish T cell tolerance to HBV. The liver environment has generally been considered tolerogenic, plausibly to avoid detrimental immune reaction to gut-derived microorganisms and xenobiotics (15, 16). The cellular and molecular immunoregulatory mechanisms behind this long-standing notion, and especially its implications on HBV clinical outcomes, are beginning to be understood.

In this mini-review, we summarize the current understanding of immunological factors deemed to contribute to T cell dysfunction in the liver. A full appreciation of the mechanisms behind intrahepatic T cell dysfunction is essential to develop a ‘cure’ for CHB and liver cancer by immune reinvigoration.

## T Cell Dysfunction During HBV Infection

Several human and animal studies have sought to define the frequency, phenotype, and function of HBV-specific CD8+ T cells to compare and contrast these features between acute HBV resolvers and CHB patients (11, 14, 17–20). There is consensus that in CHB, effector CD8+ T cells show multiple levels of an ‘exhausted’ phenotype, i.e., markedly reduced capacity to proliferate, produce IFN*γ*, IL-2, TNFα, granzymes, or perforins (9, 11, 18, 21). Characteristically low frequencies of HBV-specific CD8+ T cells are often recorded in CHB patients than in acute resolvers (14, 17, 18). Low CD8+ T cell numbers could be due to either poor expansion or increased clonal deletion. Frequency alone may not be of absolute importance over the function and breadth of the T cell population (11, 14, 20). A higher frequency of functional but partially exhausted CD8+ T cell with a CD127+ PD1+ phenotype was previously described in inactive CHB carriers. A subset showing more profound exhaustion displayed the CD127- PD1+ phenotype (20). Recently, acute resolvers were shown to have a multi-specific T cell repertoire covering HBV core, polymerase, and envelope epitopes in all the study participants. By contrast, a fraction of the CHB patients in the same study had T cells against HBV core and polymerase, with none against the HBV envelope (11). Similarly, HBV core and polymerase but not envelope specific CD8+ T cells were found in peripheral circulation in CHB patients in an independent study (20). The absence of envelope-specific CD8+ T cells is thought to reflect clonal deletion, as envelope-specific CD8+ T cells presumably become hyper-responsive to the relatively abundant hepatitis B surface antigen (HBsAg). However, caution should be exercised in interpreting these findings since only a few CD8+ T cell epitopes have been tested for each antigen. Besides the quantities of the cognate antigen, differences in antigen processing in the liver may also affect the qualitative and quantitative features of HBV-specific CD8+ T cells.

## Mechanisms of HBV-Specific CD8+ T Cell Dysfunction

**Figure 1:**
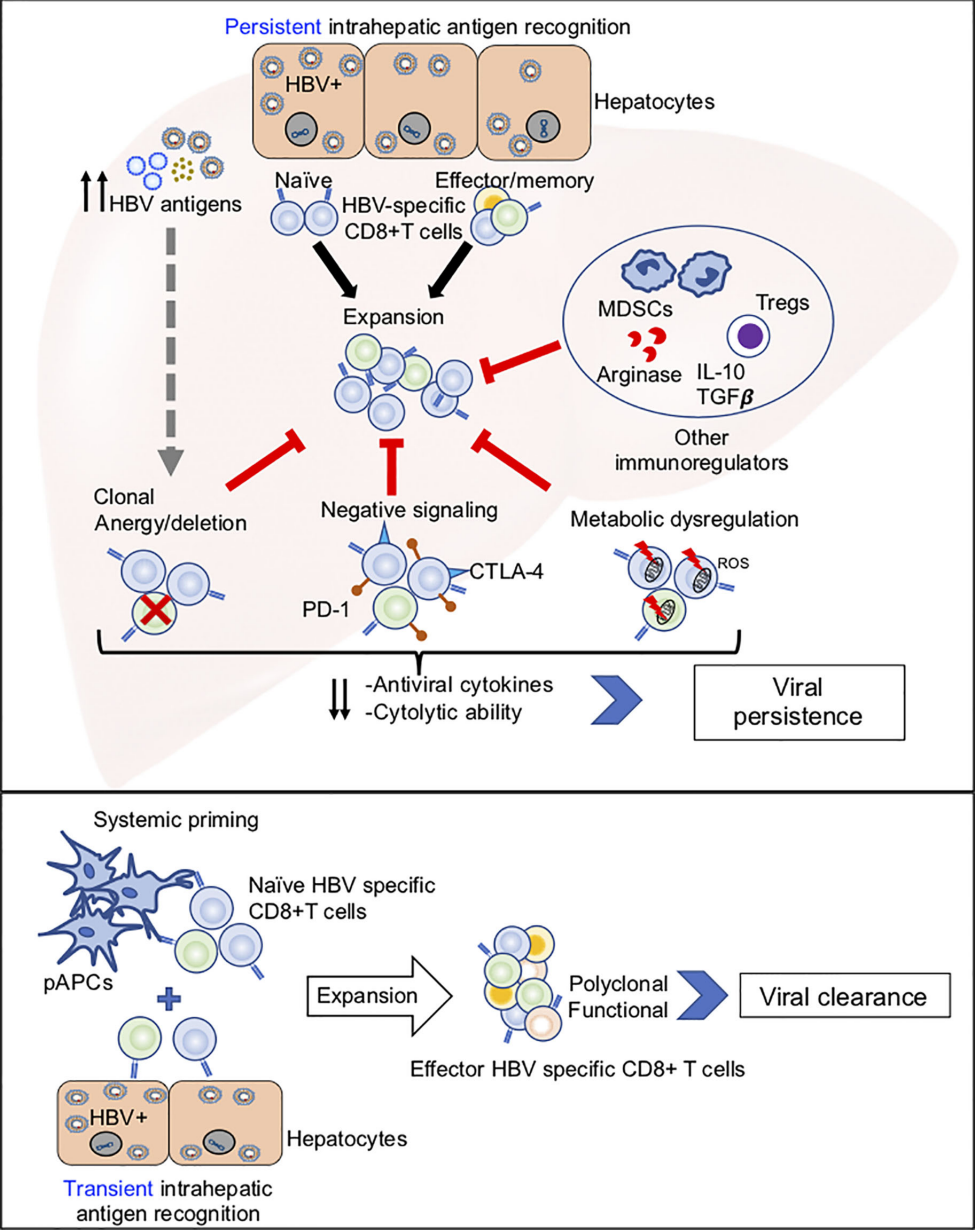
Potential mechanisms of HBV-specific CD8+ T cell dysfunction. Top panel: Illustration of how persistent antigen recognition, predominantly by HBV infected hepatocytes, results in dysfunctional HBV-specific CD8+ T cells that fail to clear infection. Bottom panel: Illustration of how systemic and hepatic antigen recognition may cooperatively trigger robust HBV-specific CD8+ T cell responses that result in viral clearance.

Potential mechanisms of HBV-specific CD8+ T cell dysfunction are summarized in **Figure 1**, and discussed below.

### Immunoregulatory Mediators in Liver Tolerance

Early studies on MHC mismatched liver transplants in animal models established the liver as a tolerogenic organ (15). Furthermore, studies in the 1970s already showed that soluble human and rat liver extracts inhibit T cell activation and DNA synthesis (22, 23). These extracts are now known to have been arginase whose high expression levels in the liver and peripheral blood have been associated with antigen nonspecific impairment of T cells in both acute and chronic HBV patients (24, 25). Hepatic necroinflammation may exacerbate the release of these enzymes by hepatic cytolysis. Plausibly, the observed high arginase levels during acute HBV infection serve as part of a regulatory feedback loop to minimize liver damage (24). Similar immunosuppressive enzymes that deplete metabolites required for the proliferation and maintenance of functional T cells, such as the tryptophan-depleting indoleamine-2-3deoxygenase (IDO) have also since been identified (15, 25, 26). These enzymes are secreted by various myeloid immune cells, including monocytic and granulocytic myeloid-derived suppressor cells (MDSCs) that have been found to be enriched in CHB patients (26–28). Interestingly, a recent study by Yang et al. reported that monocytic MDSCs (mMDSCs) were differentially upregulated in HBeAg-positive CHB patients. HBeAg was then shown to trigger mMDSC expansion that led to the IDO-mediated suppression of CD8+ T cell responses *in vitro* (28). Although the mechanism of HBeAg-induced mMDSCs expansion remains to be elucidated, this report, supplementary to the previous reports on HBeAg tolerogenicity (29, 30), suggests a novel targetable way by which HBV exploits nonspecific immunosuppressive effects to maintain liver persistence.

Other liver cell populations such as dendritic cells (DCs), liver sinusoidal endothelial cells (LSECs) hepatic stellate cells (HSCs) may also contribute towards T cell tolerance in the liver by several mechanisms that include: (i) IFN*γ*-dependent production of the soluble factors like IDO, arginase (31) (ii) activation of T regulatory cells (Tregs) *via* the expression of anti-inflammatory cytokines such as interleukin-10 (IL10) and transforming growth factor-beta (TGFβ) (15, 16, 32), (iii) upregulation of co-inhibitory receptor ligands, particularly PD-L1 that leads to T cell exhaustion in a positive feedback cycle with IL10 and TGFβ1 (32, 33) (iv) Expression of cell killing ligands like FasL and TRAIL (34). Notably, liver DCs have been described as immature and dysfunctional compared to peripheral DCs (35). However, this remains controversial in CHB because some studies don’t report any such difference (36).

### Negative Signaling Mechanisms

Exhausted CD8+ T cells exhibit reduced effector function often in association with upregulation of co-inhibitory receptors such as PD-1, cytotoxic T-lymphocyte associated antigen 4 (CTLA-4), T-cell immunoglobulin and mucin domain-containing protein (Tim-3) (37–41). Of these, PD1-PD-L1 interactions have so far received the greatest attention as a target for tumor immune therapy. Ligation of PD-L1 to PD-1 receptors on T cells impairs downstream TCR signaling to inhibit their immune activation (33). A brief overview of how PD-1 expression is regulated in general is given by Bally et al. (42). The role of PD-1-PD-L1 in HBV-specific CD8+ T cell dysfunction (37, 38, 43–46) has been intensively investigated. Sustained PD-1upregulation is correlated with HBV-specific T cell dysfunction during CHB (18, 43) and PD-L1 expression on peripheral blood was shown to be upregulated in CHB patients (47). PD-L1 expression could also be induced on hepatocytes by type I and type II interferons (48). Anti-PD-L1 treatment on CHB patient-derived peripheral and intrahepatic HBV-specific CD8+ T cells enhanced IFNγ expression *in vitro* (38, 44), suggesting the immune restoration potential of PD-1 blockade. However, promising results of the *in vitro* studies do not necessarily assure therapeutic value *in vivo*. In HBV transgenic mice, antibody blockade, as well as genetic removal of PD-1 signaling, increased the frequency of HBV-specific CD8+ T cells, but the majority of HBV-specific CD8+ T cells remained dysfunctional (37). Importantly, a recent clinical study by Gane et al. showed that treatment of HBeAg negative CHB patients with a single dose of the PD-1 antibody Nivolumab resulted in modest HBsAg reduction within 24 weeks without any adverse events (46), and only one out of 10 patients exhibited HBsAg seroconversion and strong induction of HBV-specific CD8+ T cell responses. While the results were encouraging, the therapeutic impact of PD-1 signaling blockade was rather marginal. The data raises the possibility that other yet unknown co-regulatory molecules are present to suppress HBV-specific CD8+ T cell responses. Simultaneous blockade of multiple inhibitory receptors seems to improve therapeutic potential. In vivo co-blockade of PD-1/LAG-3 and PD-1/Tim-3 during LCMV infection synergistically enhanced CD8+ T cell responses (49, 50). Dual PD-1/CTLA-4 pathway blockade showed similar synergism in partially reversing HBV-specific CD8+ T cell exhaustion *in vitro* (51) There is a paucity of data on the impact of multiple target blockade in CHB, and the nature and extent of negative regulatory molecules’ co-regulation and expression may differ between patients. Personalized T cell characterization may be required for optimized treatment to reverse T cell exhaustion.

### Metabolic Dysregulation in T Cells

Metabolic reprogramming after priming is important for T cell differentiation because energy demand largely differs between naïve, effector, and memory T cells (52–54), and mitochondrial plasticity is directly linked to T cell metabolism (55). Metabolic abnormalities, such as reduced glycolysis and oxidative phosphorylation, were observed in exhausted virus-specific CD8+ T cells during the early phase of chronic lymphocytic choriomeningitis virus (LCMV) infection (56). PD-1 high HBV-specific CD8+ T cells in CHB patients were also shown to highly express the glucose transporter, Glut1, and dependent on glucose supplies (57). These changes were accompanied by increased mitochondrial size and lower mitochondrial potential. Recently, Fisicaro et al. reported extensive mitochondrial dysfunction, such as mitochondrial membrane potential depolarization and reactive oxygen species (ROS) elevation in association with upregulation of co-inhibitory receptor genes in the CD8+ T cells from CHB patients (58). More importantly, mitochondrial antioxidant treatment using mitoquinone and a piperidine-nitroxide could modestly enhance IFN*γ* production by HBV-core specific CD8+ T cells from these patients (58), indicating a potential role for ROS in CD8+ T cell exhaustion. Coordinated protein catabolism by the ubiquitin-proteasome and autophagy-lysosome systems is also essential for CD8+ T cell survival, proliferation, and memory formation (59–61). In addition, autophagy was recently shown to enable HBV-specific effector memory CD8+ T cells to reside in the liver and resist mitochondrial depolarization (62). Importantly, genes associated with ubiquitin-proteasome and autophagy-lysosome systems were also markedly downregulated in exhausted HBV-specific CD8+ T cells in CHB patients (59, 63). In vitro treatment of exhausted HBV-specific CD8+ T cells with polyphenols, such as resveratrol and oleuropein, improved autophagic influx and antiviral CD8+ T cell function (64). More recently, p53, a known negative regulator of glycolysis and an enhancer of oxidative phosphorylation (OXPHOS), was shown to be upregulated in exhausted HCV-specific CD8+ T cells from chronic HCV patients (65). Its relevance to chronic HBV infection remains to be determined because p53 was thought to be upregulated by type I interferon (IFN-I) response, which is largely absent during HBV infections. Overall, these results characterize CD8+ T cell exhaustion as a state of metabolic insufficiencies with suppressed mitochondrial respiration, glycolysis, protein degradation. These abnormalities are reminiscent of functional defects previously associated with CD8+ T cell senescence, although exhaustion and senescence are distinctly different in terms of generation, development, and metabolic and molecular regulation (63, 66).

### Intrahepatic Antigen Recognition

The tolerogenic environment in the liver likely contributes to imprinting the genetic and epigenetic signatures in the dysfunctional HBV-specific CD8+ T cells during CHB. It is important to keep it in mind, however, that efficient HBV-specific CD8+ T cell responses are induced in the majority of adult-onset HBV infections, resulting in viral clearance. Factors that determine the dichotomy presumably include traditional factors such as T cell receptor (TCR) signaling (signal 1), co-stimulatory signaling (signal 2), and cytokine signaling (signal 3).

A strong antigenic stimulus is necessary for effective CD8+ T cell responses (67). We have shown recently that the magnitude of HBV-specific CD8+ T cell responses was directly correlated with the level of early antigen expression in an animal model of transient HBV infection, i.e., hydrodynamic transfection of HBV plasmid (67). Suppression of HBV by siRNA also inhibited the expansion of HBV-specific CD8+ T cells (67), indicating the importance of strong antigen recognition for the induction of HBV-specific CD8+ T cell responses. Paradoxically, the same antigenic stimulus becomes extremely detrimental for T cell responses if it is prolonged (68–70). Indeed, HBV-specific effector memory CD8+ T cells that were generated by DNA-prime, vaccinia-boost immunization produced a large amount of IFNγ upon antigen recognition in the liver, but they lost the IFNγ producing ability almost completely within three days during which they continuously recognized antigen and express PD-1 (71). Slow blood flow in the liver sinusoid, as well as tightly packed microanatomy of the hepatic parenchyma, facilitate prolonged interaction between HBV infected hepatocytes and HBV-specific CD8+ T cells (72). Intravital imaging analysis revealed that HBV-specific CD8+ T cells were able to recognize HBV expressing hepatocytes while they were still in the sinusoid (73). Prolonged antigen recognition appears to inhibit TCR signaling partially through PD-1 signaling (74).

Antigen presentation by hepatocytes alone is probably insufficient for priming of functional HBV-specific CD8+ T cell responses. We have previously shown that HBV-specific naïve CD8+ T cells are primed by HBV-expressing hepatocytes (75). Although hepatocyte-primed naïve and memory HBV-specific CD8+ T can expand rapidly, they produce very little to no IFN*γ* and Granzyme B (75–77). The lack of functional differentiation presumably reflects the absence of co-stimulatory signaling (i.e., signal 2) as hepatocytes do not express ligands for co-stimulatory molecules. Indeed, activation of dendritic cells appears to facilitate functional differentiation of intrahepatically primed CD8+ T cells (75, 76). In addition, expression of a co-stimulatory molecule OX40 expression by CD4 T cells and its ligand OX40L by hepatic innate immune cells were shown to be pivotal in determining HBV immunity in an HBsAg transgenic mouse model (78).

Recently, we and others characterized genetic signatures of intrahepatic T cell priming. Similar to HBV-specific CD8+ T cells in CHB patients, the intrahepatically primed, dysfunctional CD8+ T cells showed upregulation of inhibitory molecules PD-1, Lag 3, and Tim-3, together with enrichment in binding sites for the transcription factors AP-1, NFAT, NR4A, OCT, TCF, and EGR (79). Interestingly, NR4A has been implicated in T cell exhaustion that limits CAR-T cell-based immunotherapy in solid tumors and LCMV infection (80, 81). In stark contrast to exhausted CD8+ T cells during LCMV infection, genes related to IFN-I signaling activation were downregulated in intrahepatically primed T cells. Importantly, strong stimulation of IFN-I signaling in the liver enhanced T cell responses (82), suggesting that IFN-Is indeed provide the third signal (signal 3) that complements the TCR signal (signal 1) and co-stimulatory signal (signal 2). It should be noted, however, that the same IFN-I signaling could suppress HBV-specific CD8+ T cell responses by reducing antigen expression levels during the early phase of T cell priming (67), a phenomenon recently highlighted in the development of RNA vaccines (83).

## Perspectives

Recent advances in unbiased deep sequencing and other genetic analysis methods have accelerated the delineation of CD8+ T cell dysfunction in the liver, providing numerous targets to test for novel immunotherapies against CHB. It would be now important to determine whether the functionalities of highly exhausted T cells are reversible. Even more crucial is to establish an ideal animal model for evaluating the therapeutic value of each target. Several mouse models have been used to study HBV-specific CD8+ T cell responses during transient and persistent antigen expression. The advantages and disadvantages of each mouse model have been described elsewhere (84, 85). While these models provided useful information on T cell responses, none of them mimics a bona fide HBV infection. It is therefore essentially impossible to assess the extent to which chronically infected individuals can tolerate the restoration of HBV-specific CD8+ T cell responses, as the expansion of functional CD8+ T cells likely cause hepatitis. In this regard, antigen suppression should be incorporated in the immune restoration approach to mitigate the risk of uncontrolled T cell expansion.

## Conclusion

Given the stability of cccDNA, invigoration of HBV-specific CD8+ T cells remains one of the most viable approaches to cure CHB. Therefore, delineation of the key pathways and processes that underline HBV-specific CD8+ T cell dysfunction T cell is an important research goal to develop effective HBV immunotherapies.

## Author Contributions

MI conceived the outline of the manuscript. IB and MI wrote the original manuscript. IB and MI selected the references. IB prepared the Figure. KK reviewed the manuscript. All authors contributed to the article and approved the submitted version.

## Funding

This work was supported by grants-in-aid from the Ministry of Education, Cultures, Sports, Science, and Technology, Japan: From Japan Society for Promotion of Science (KAKENHI) under grants 20K08313 (to MI); and from the Research Program on Hepatitis from the Japan Agency for Medical Research and Development (AMED) under grants 21fk0310106s0405 (to MI), 21fk0310101h1205 (to MI), and 21fk0310103j8005 (to MI).

## Conflict of Interest

The authors declare that the research was conducted in the absence of any commercial or financial relationships that could be construed as a potential conflict of interest.

## Publisher’s Note

All claims expressed in this article are solely those of the authors and do not necessarily represent those of their affiliated organizations, or those of the publisher, the editors and the reviewers. Any product that may be evaluated in this article, or claim that may be made by its manufacturer, is not guaranteed or endorsed by the publisher.
